# Topical Losartan in Ophthalmology: Rationale, Experimental and Clinical Evidence, and Emerging Clinical Applications

**DOI:** 10.3390/jcm15041354

**Published:** 2026-02-09

**Authors:** Barbara Burgos-Blasco, Mercedes Molero-Senosiain, Pilar Perez-Garcia, Mayte Ariño-Gutierrez, Pedro Arriola-Villalobos, David Diaz-Valle

**Affiliations:** 1 Servicio Oftalmologia, Hospital Clínico San Carlos, Instituto de Investigación Sanitaria del Hospital Clínico San Carlos (IdISSC), 28040 Madrid, Spain; 2 Departamento de Oftalmología, Inmunología y ORL, Facultad de Medicina, Universidad Complutense de Madrid, 28040 Madrid, Spain

**Keywords:** cornea, haze, fibrosis, topical, losartan, angiotensin

## Abstract

Corneal fibrosis remains a major cause of visual morbidity, with transforming growth factor-β (TGF-β) signaling playing a central role in this process. Losartan, an angiotensin II type 1 receptor blocker widely used systemically for cardiovascular indications, has recently attracted interest in ophthalmology due to its antifibrotic properties through indirect inhibition of TGF-β signaling. In recent years, increasing experimental and early clinical evidence has suggested that topical ophthalmic formulations of losartan may attenuate corneal fibrosis following diverse injuries such as descemetorhexis, alkali burns, and photorefractive keratectomy-related injury. Topical losartan represents a promising, non-cytotoxic antifibrotic strategy in ophthalmology, although human evidence is limited and further randomized controlled clinical trials are required to define its clinical efficacy, optimal indications, timing, posology, formulations, and long-term safety. This review summarizes the biological rationale for the use of topical losartan in ophthalmology, including its molecular mechanisms of action, pharmacologic considerations, and safety profile. We critically review preclinical studies in corneal models, as well as emerging clinical applications.

## 1. Introduction

Corneal fibrosis represents a significant challenge in ophthalmology, contributing to vision impairment and compromised surgical outcomes, such as refractive surgeries and corneal transplants. Current antifibrotic strategies are largely aimed at preventing the development of corneal haze rather than reversing established fibrosis. These include intraoperative measures, such as mitomycin-C (MMC), and postoperative control of inflammation with topical corticosteroids or immunomodulatory agents such as cyclosporine. While these approaches can be effective in reducing early scarring, their long-term use is limited by well-recognized adverse effects, including increased intraocular pressure, cataract formation, delayed wound healing, and epithelial toxicity. Moreover, none of these treatments reliably reverse established stromal fibrosis once it has developed. These limitations highlight the urgent need for new and more selective antifibrotic therapies that can offer effective outcomes without the adverse effects associated with traditional treatments.

Losartan, an angiotensin II receptor blocker, has gained attention in recent years as its antifibrotic properties have become increasingly recognized. Originally developed as an oral arterial hypotensor, losartan has shown significant potential in modulating fibrosis in various organs, including the heart, kidneys, and liver. More recently, research has suggested that losartan may also have a role in ophthalmology due to its role in the modulation of transforming growth factor-β (TGF-β) signaling, a pathway known to drive corneal fibrosis. In experimental models, topically applied losartan has been shown to attenuate stromal fibrotic haze following various corneal injuries and surgical procedures, an effect attributed to its ability, when administered topically, to achieve direct local inhibition of fibrotic signaling within the cornea; these findings have been supported by emerging case reports and small case series in humans. As a result, the topical use of losartan could provide a promising adjunct to existing therapies, offering a more selective and safer approach to managing corneal fibrosis.

This review aims to explore the current understanding of losartan’s mechanism of action in the context of ocular fibrosis, its potential role in preventing and treating corneal fibrosis, and the emerging evidence supporting its topical use. We will examine preclinical studies, emerging clinical data, and the potential advantages and challenges of using losartan in fibrosis management.

### Search Strategy and Selection Criteria

We conducted a comprehensive literature search to identify all relevant studies on the clinical and preclinical applications of topical losartan for corneal fibrosis. The main databases searched included PubMed/MEDLINE, Embase, and the Cochrane Library. The search was restricted to studies in both animals and humans published in English with no specific time frame. Core search terms included combinations of: “losartan,” “topical,” “drops,” “cornea,” “fibrosis,” and “haze.”

Inclusion criteria were: (1) peer-reviewed publications reporting the use of topical losartan for corneal haze or fibrosis; (2) original articles (prospective or retrospective), case series, animal studies and randomized trials, systematic reviews and meta-analyses. Exclusion criteria were: conference abstracts without full text, narrative reviews and non-peer-reviewed material.

Study selection was performed independently by two authors who reviewed titles and abstracts, followed by full-text evaluation of potentially eligible studies. While no formal PRISMA protocol was registered, we emphasized methodological rigor in including all related publications. The evidence base primarily consists of retrospective studies, case series, and animal studies; these limitations are acknowledged in the discussion. While a structured search strategy was applied to ensure rigor and transparency, it does not follow systematic review or meta-analysis methodology, and therefore does not claim exhaustive completeness.

## 2. The Role of TGF-β Signaling in Corneal Fibrosis

The corneal wound healing response following epithelial–stromal injury comprises a complex sequence of events that typically promotes restoration of normal stromal architecture and function. However, in certain circumstances, dysregulated healing processes result in compromised corneal transparency. Although the corneal wound healing cascade has been shown to be regulated by several growth factors, TGF-β isoforms are the most significant regulators of corneal fibrosis through their effects on myofibroblasts and the increase in collagen type IV production [1,2].

After corneal epithelial–stromal injuries, different mechanisms occur as part of the corneal wound healing process. TGF-β is rapidly released into the extracellular environment, initiating multiple cellular responses that are critical for effective wound repair. TGF-β promotes corneal epithelial cell migration and proliferation, both of which are required for re-epithelialization. In addition, TGF-β stimulates keratocyte proliferation and their differentiation into myofibroblasts. These myofibroblasts subsequently regulate the deposition of a provisional extracellular matrix within the wound area, including collagen, fibronectin, laminin, proteoglycans, hyaluronan, matrix metalloproteinases (MMPs), and tissue inhibitors of metalloproteinases (TIMPs), all of which play essential roles in corneal healing. The emergence of myofibroblasts has been recognized as a pivotal determinant in the development of corneal stromal opacity or fibrosis. These cells, which are intrinsically opaque due to reduced corneal crystallin expression, synthesize excessive amounts of disorganized extracellular matrix once established within the anterior stroma, ultimately leading to stromal haze [1,2].

The epithelial basement membrane functions as a critical regulatory barrier for these molecules within the cornea. Disruption of the epithelial basement membrane allows increased penetration of TGF-β into the stroma, where it leads to loss of corneal transparency. Persistent TGF-β signaling is a central driver of chronic corneal fibrosis observed after refractive surgery, keratoplasty, and severe corneal trauma [1].

For transparency to be restored, TGF-β deprivation is needed for myofibroblasts to undergo apoptosis and allow keratocytes to reoccupy the anterior stroma and absorb the disordered extracellular matrix. Persistent or dysregulated activation of this pathway leads to chronic fibrosis characterized by excessive extracellular matrix deposition, tissue stiffening, and loss of normal architecture [1,3,4]. Thus, precise control of TGF-β activity is fundamental for preserving corneal transparency and optimal visual function after injury [2,5].

In this framework, strategies aimed at modulating, rather than completely inhibiting, TGF-β activity may offer a means to attenuate pathological fibrosis while preserving normal wound healing. This concept provides a strong biological rationale for exploring agents such as losartan, which indirectly modulate TGF-β signaling, as novel antifibrotic therapies in ophthalmology.

## 3. Pharmacology of Losartan

Losartan is generally used to treat arterial hypertension and was approved for medical use in the United States in 1995. In 2020, it was the ninth most commonly prescribed medication in the United States, with more than 54 million prescriptions. Besides hypertension, it is also used in other conditions, including diabetic kidney disease, heart failure, and left ventricular hypertrophy [6,7].

Losartan exerts its effects through selective, competitive and reversible blockade of the angiotensin II type 1 (AT1) receptor, thereby counteracting the actions of angiotensin II, a potent vasoconstrictor and the principal vasoactive effector hormone of the renin–angiotensin system. Angiotensin II plays a central role in the pathophysiology of arterial hypertension by promoting sodium and fluid retention and stimulating vascular smooth muscle cell growth. By inhibiting the binding of angiotensin II to AT1 receptors, which are widely expressed in tissues such as vascular smooth muscle, kidneys, heart, and glandular structures, losartan attenuates these downstream effects [6].

Beyond its hemodynamic actions, angiotensin II stimulates TGF-β expression and upregulates receptors for TGF-β in some tissues by various mechanisms. Furthermore, evidence suggests that other components of the renin–angiotensin–aldosterone system including angiotensin III, renin, and aldosterone also activate the TGF-β system. As direct modulation of the TGF-β system is not yet feasible in humans, angiotensin-converting enzyme inhibitors and AT1-receptor blockers are currently the most potential drugs to interfere with this angiotensin II-mediated TGF-β expression. Losartan is thus thought to exert its antifibrotic effects by modulating the TGF-β signaling pathway, a crucial mediator in corneal fibrosis [8,9,10].

Oral losartan is generally well tolerated. In the Losartan Intervention For Endpoint reduction in hypertension study (LIFE) study, a randomised trial to investigate cardiovascular morbidity and mortality against atenolol, the most common adverse event in losartan recipients was dizziness. Other common adverse events in losartan recipients (occurring in 9.9–12.3% of patients) included back pain, lower extremity oedema, chest pain and dyspnoea [11].

## 4. Rationale for Topical Ophthalmic Use of Losartan

The use of topical losartan for corneal fibrosis might offer several potential advantages in the context of ocular conditions, as effective treatment requires local modulation of fibrotic signaling within an avascular tissue.

Topical administration has a clear advantage in terms of direct delivery to the site of action. Unlike systemic treatments, which involve drug absorption, metabolism, and distribution throughout the body, topical ophthalmic therapies allow for targeted delivery to the eye. This localized treatment reduces the need for high systemic doses, thus minimizing systemic exposure and the associated side effects typically seen with oral or systemic treatment and offering a more direct approach and potentially quicker therapeutic outcomes. Systemic administration of losartan is associated with common side effects such as dizziness, hyperkalemia, or hypotension due to its effects on blood pressure and renal function. Therefore, this topical approach could be particularly beneficial in patients who are already on other antihypertensive medications or those with contraindications to systemic losartan.

Despite its theoretical advantages, formulation of topical losartan for ocular use presents challenges related to corneal penetration. The corneal epithelium, due to its lipid bilayer structure, constitutes a major barrier to drug diffusion into the stroma, where fibrotic remodeling occurs. It can also increase toxicity in cases with an epithelial defect [12,13].

Current antifibrotic therapies for corneal haze and fibrosis include corticosteroids, MMC, 5-fluorouracil (5FU), and anti-VEGF agents. While these treatments have been effective in certain contexts, they each have limitations. Corticosteroids, for example, carry the risk of elevated intraocular pressure and cataract formation with prolonged use. MMC and 5FU, while effective in preventing scarring, are cytotoxic agents that can damage surrounding tissues and delay healing. Anti-VEGF therapies, which target vascular endothelial growth factors, are typically used in neovascular conditions rather than fibrosis.

In contrast, losartan offers several potential advantages. It is not cytotoxic and it specifically targets the molecular pathways involved in fibrosis without affecting the normal healing process. By modulating TGF-β signaling, losartan could reduce collagen type IV deposition and limit the differentiation of keratocytes in fibroblasts and their maturation to myofibroblasts, key drivers of corneal scarring. This targeted mechanism could offer a more refined and safer alternative to the broader action of steroids and other cytotoxic agents, especially in patients who require long-term management of corneal fibrosis.

Based on this, Dr Wilson hypothesized possible indications include corneal scarring associated with corneal trauma, chemical burns, infections, surgical complications, persistent epithelial defects, conjunctival fibrotic diseases and TGFBI-related corneal dystrophies (Reis–Bucklers corneal dystrophy, lattice corneal dystrophy type 1, and granular corneal dystrophies type 1 and type 2) [14].

## 5. Preclinical Evidence

Animal models were initially used to evaluate the efficacy and safety of topical losartan in modulating corneal wound healing, specifically targeting the prevention and treatment of corneal scarring and fibrosis (Table 1) [13]. Experimental models, predominantly in rabbits, have demonstrated that topical losartan can reduce stromal fibrosis after various corneal injuries, including Descemetorhexis without endothelial replacement (Descemet Stripping Only—DSO), alkali burns, incisions, phototherapeutic keratectomy (PTK), and photorefractive keratectomy (PRK). Importantly, losartan has been shown to penetrate the full thickness of the cornea, allowing it to address both anterior and posterior stromal fibrosis [15].

In a posterior-fibrosis paradigm (8-mm DSO), topical losartan (0.4 mg/mL; 50 μL; six times daily for one month) significantly reduced central opacity and peripheral scarring, and Smooth Muscle Actin (α-SMA)-positive posterior fibrosis, whereas high-dose oral losartan (5 mg/kg three times daily) was ineffective. Notably, topical treatment also reduced pathological stromal type IV collagen deposition, supporting in vivo modulation of TGF-β-mediated fibrotic signalling [16].

In severe full-thickness alkali burn injury (NaOH one minute exposure), prophylactic treatment initiated at the same time as the injury (losartan 0.2 mg/mL; six times daily and prednisolone acetate 1% for one month) demonstrated that each agent independently reduced corneal opacity compared with vehicle. Combined treatment resulted in the most consistent attenuation of opacity intensity and α-SMA-positive myofibroblast accumulation, along with relative restoration of keratocan-positive keratocytes in the anterior and mid-stroma. In the same model, dual therapy also prevented central corneal neovascularisation, indicating superior antifibrotic and antiangiogenic efficacy in this aggressive injury model [17].

Therapeutic (rather than prophylactic) use has also been explored. In established fibrosis (defined as that at one month after alkali burn and allowed to mature for that time prior to treatment), topical losartan (0.8 mg/mL; six times daily) reduced stromal myofibroblast density and increased stromal cell apoptosis at both one week and one month, although the short-interval change in the level of opacity only showed a non-significant trend towards improvement [20].

Not all contexts translate myofibroblast suppression into clinically evident clarity within short follow-up. After blast-simulating irregular PTK designed to perpetuate epithelial basement membrane dysfunction, topical losartan (0.8 mg/mL; six times daily for six weeks) significantly reduced anterior stromal α-SMA expression but did not improve central corneal opacity or alter stromal keratocan, vimentin, TGF-β1, or collagen type IV levels [19]. Similarly, in nearly full-thickness radial incisions closed with a suture, topical losartan (0.8 mg/mL; six times daily for one month) did not impair wound closure and did not significantly change incision-associated opacity but did significantly reduce the density of stromal myofibroblasts adjacent to the wound [21].

Complementary rabbit studies elucidate key biological mechanisms underlying post-PRK fibrosis. Early persistent epithelial defects promote rapid anterior stromal myofibroblast accumulation through impaired epithelial basement membrane regeneration and sustained TGF-β signalling [22]. In parallel, in a high-correction PRK model (–9.00D), topical losartan (0.2 mg/mL; six times daily for one month) significantly reduced central corneal opacity, subepithelial myofibroblast generation, and collagen type IV deposition, supporting targeted modulation of fibrotic wound-healing pathways [18].

## 6. Emerging Clinical Evidence

Available human data on topical losartan remains scarce and limited to a small number of case reports and case series, precluding definitive conclusions regarding efficacy. To date, only six publications have reported clinical outcomes following topical losartan use in humans, collectively encompassing results from 30 patients (Table 2) [12,23,24,25,26,27].

Overall, the most frequently reported indications for topical losartan include corneal haze following refractive surgery, either alone or combined with corneal cross-linking, as well as post-infectious corneal leukomas. Less commonly reported and more anecdotal indications include stromal fibrosis associated with corneal hydrops or chronic corneal edema, and corneal scarring secondary to adenoviral conjunctivitis, chemical burns, interstitial keratitis, herpetic keratitis and pterygium surgery.

Interpretation of the published data is complicated by several methodological limitations. In many reports, the interval between the inciting ocular insult (surgery, infection, or herpetic reactivation) and the initiation of topical losartan is not specified. Similarly, the duration of the corneal leukoma and whether fibrosis was clinically stable at treatment onset are often unclear. These factors may bias efficacy estimates toward more favorable outcomes, as spontaneous corneal remodeling and gradual clearing can occur over time independently of therapeutic intervention.

To date, dosing has not been standardized across human studies. Concentrations used in clinical reports have largely been guided by preclinical safety data and early clinical experience. While most clinical studies employed topical losartan at a concentration of 0.8 mg/mL [12,24,25,26,27], the largest published case series used a higher concentration of 1 mg/mL [23]. This variation likely reflects pragmatic compounding considerations and extrapolation from preclinical protocols rather than evidence of superior efficacy, as no dose–response comparisons have been conducted. Although different vehicles have been explored in preclinical studies, human reports have predominantly used topical losartan compounded in balanced salt solution from USP-grade losartan potassium powder. Importantly, preparation from crushed oral tablets is discouraged due to the presence of excipients that may be potentially toxic to the ocular surface. Most reports further emphasize that sterilization of the final solution should be performed using 0.2-µm filtration rather than heat-based methods prior to clinical use [12,24].

In most reported cases, topical losartan was administered at a high-frequency dosing regimen of four to six times daily, underscoring the apparent need for sustained stromal exposure to achieve antifibrotic effects. Treatment duration varied widely according to clinical response, ranging from as short as 2–4 weeks in milder presentations to 3–9 months in more severe or chronic stromal scarring. Prolonged treatment until clinical stabilization has been explicitly advocated in expert guidance and applied in selected case series, based on the proposed need to allow epithelial basement membrane regeneration and sustained suppression of myofibroblast survival [23]. However, no studies to date have systematically evaluated outcomes following treatment discontinuation or the durability of these effects over time.

In terms of efficacy, most human reports describe a progressive reduction in corneal haze and stromal opacity, with clinical improvement typically observed within the first three months of therapy. Visual outcomes generally parallel structural changes, with reported gains in uncorrected and corrected distance visual acuity ranging from modest improvements of one to two Snellen lines to more substantial recovery in selected cases. Nonetheless, it should be acknowledged that the available evidence may be subject to publication bias, as most published reports describe favorable outcomes, while cases with limited or no response may be underreported. This further limits the ability to draw definitive conclusions regarding treatment efficacy.

Comparison of the two main published case series reveals divergent efficacy outcomes, suggesting that not all patients respond uniformly to topical losartan. In the prospective series by Domene-Hickman et al. [23] statistically significant improvements in both uncorrected distance visual acuity and objective corneal densitometry parameters were observed at three months, with nearly half of treated eyes gaining two or more Snellen lines. In contrast, the series by Burgos-Blasco & Moloney [24] demonstrated a more heterogeneous response, with no statistically significant changes in visual acuity or densitometry over six months, despite a trend toward reduced corneal opacity. These discrepancies may relate to differences in dosing frequency, treatment duration, sample size, and heterogeneity in etiology and fibrosis chronicity, highlighting the current inconsistency of human efficacy data. Notably, lower dosing frequencies (e.g., four times daily) have been associated with more variable outcomes, suggesting a possible dose–response relationship that remains to be formally defined.

Domene-Hickman et al. [23] proposed a simple, clinically interpretable predictive score derived from machine-learning analysis, identifying baseline uncorrected distance visual acuity as the strongest predictor of visual response to topical losartan, whereas baseline corneal densitometry showed limited predictive value. Although internally validated, this scoring system remains exploratory and requires external validation before clinical adoption.

## 7. Safety Considerations and Knowledge Gaps

Topical losartan eye drops have demonstrated a favourable safety profile in animal studies, the main safety concern being dose-dependent corneal epithelial toxicity. When the corneal epithelium is absent, stromal exposure increases, amplifying both therapeutic activity and toxicity risk. Concentrations above 0.8 mg/mL have been associated with delayed epithelial healing, persistent epithelial defects, and increased opacity; therefore, lower concentrations (0.2 mg/mL) are recommended until epithelial closure, after which escalation may be considered. Current evidence indicates that appropriately dosed topical losartan does not compromise stromal remodelling or wound integrity. However, the long-term and full impact of sustained TGF-β modulation on corneal biomechanics, nerve regeneration, and epithelial–stromal interactions remain insufficiently understood [12,13].

In corneas with an intact epithelium, 0.8 mg/mL administered six times daily appears safe throughout treatment. No clinically relevant changes in intraocular pressure or corneal topography have been reported, with no documented allergy or hypersensitivity.

In humans, topical losartan has also demonstrated a favorable tolerability profile. Across published case reports and series, no serious ocular adverse events have been reported, even with prolonged high-frequency topical dosing [12]. Caution has been advised in the presence of epithelial instability, with some authors recommending temporary dose reduction in such cases.

Several translational challenges persist. Topical losartan is currently available exclusively as a compounded formulation, with variability in vehicle composition, pH, osmolarity, and stability, thereby limiting reproducibility and comparability across studies. From a regulatory perspective, its off-label status, absence of Good Manufacturing Practice-grade commercial formulations, and lack of large, controlled trials constitute barriers to widespread clinical adoption [12,13,24].

## 8. Conclusions

The theoretical rationale for the use of topical losartan in the prevention or treatment of corneal fibrosis is biologically plausible and may represent a promising therapeutic tool. However, despite encouraging preliminary findings, the current evidence remains limited and largely exploratory, and no conclusive clinical studies have definitively demonstrated its efficacy in corneal applications.

Future research should primarily focus on generating robust preclinical and clinical evidence to validate this therapeutic concept. Randomized clinical trials are needed to assess safety, tolerability, and potential signals of efficacy before any conclusions regarding clinical benefit can be drawn. Future studies should aim to clarify the indications, optimal dosage, timing, frequency, and duration of topical losartan treatment, as these factors may significantly influence therapeutic outcomes. Additionally, the potential for synergistic effects when combined with other antifibrotic agents, anti-inflammatory therapies or epithelial regenerating agents also holds promise. Exploring combination therapies could enhance the therapeutic outcomes for patients with more severe forms of corneal haze or fibrosis.

In conclusion, while the preliminary results regarding topical losartan are encouraging, further research is essential to define its use in clinical practice. Topical losartan should currently be regarded as an experimental and hypothesis-generating approach rather than an evidence-based treatment for corneal haze or fibrosis. While its proposed mechanism suggests potential utility, substantial gaps in knowledge remain. Addressing these gaps through rigorous translational and clinical research is necessary before topical losartan can be implemented as a therapeutic option in routine ophthalmic practice.

## Figures and Tables

**Table 1:** Animal studies on topical losartan.

Study (Year)	Animal/Eyes (N)	Indication	Treatment Groups	Losartan Formulation & Dose	Treatment Duration	Outcomes
Sampaio LP et al., *Exp Eye Res.* 2022. [16]	28 New Zealand white rabbits. 12 sham control surgery 16 DSO	Corneal scarring fibrosis and collagen type IV deposition after Descemet’s membrane–endothelial excision	1. Sham control surgery: 4 Topical, 4 oral, 4 vehicle 2. DSO: 4 Topical, 4 oral, 4 combination, 4 combination vehicle	topical losartan (0.4 mg/mL; 50 μL; 6×/day vs. oral losartan (5 mg/kg 3×/day)	1 month	Reduced central opacity and peripheral scarring IHC: Reduced α-SMA-positive posterior fibrosis The oral dose was ineffective
Sampaio LP et al., *Transl Vis Sci Technol.* 2022. [17]	16 New Zealand White rabbits	Corneal scarring fibrosis secondary to alkali burn injury	4 Topical losartan, 4 prednisolone acetate 1%, 4 combination, 4 BSS	Topical losartan 0.2 mg/mL; six times daily +/− prednisolone acetate 1%	1 month	Attenuation of opacity intensity IHC: reduced α-SMA-positive myofibroblast burden, with relative restoration of keratocan-positive keratocytes in the anterior and mid-stroma Dual therapy prevented central corneal neovascularisation
Sampaio LP et al., *J Refract Surg.* 2022. [18]	12 New Zealand White rabbits	Late haze (scarring fibrosis) after high-refraction PRK	6 Topical losartan vs. 6 vehicle	0.2 mg/mL 6×/day	1 month	Reduced central corneal opacity IHC: Reduced subepithelial myofibroblast generation and collagen type IV deposition
Sampaio LP et al., *Transl Vis Sci Technol.* 2023. [19]	12 New Zealand White rabbits	Surface blast-simulating irregular PTK to inhibit epithelial basement membrane regeneration.	6 Topical losartan vs. 6 BSS	0.8 mg/mL; 6×/day	6 weeks	Did not improve central corneal opacity IHC: Reduced anterior stromal α-SMA expression Did not alter stromal keratocan, vimentin, TGF-β1, or collagen type IV levels.
Villabona Martinez V et al., *Transl Vis Sci Technol.* 2024. [20]	24 New Zealand White rabbits	Established corneal fibrosis one month without treatment after alkali burn injury	12 Topical losartan vs. 12 vehicle BSS 6 corneas were analyzed at 1 week or 1 month in each group	0.8 mg/mL; 6×/day	1 month	Opacity trend to improve slightly over 3 weeks IHC: Decreased stromal myofibroblast density Increased stromal cell apoptosis of myofibroblasts
Villabona Martinez V et al., *Cornea.* 2024. [21]	12 New Zealand White rabbits	Acute incisions (0.35 mm deep radial into the limbus and sutured with nylon 10.0)	6 Topical losartan vs. 6 vehicle (BSS)	0.8 mg/mL; 6×/day	1 month	Did not impair wound closure Did not change incision-associated opacity

α-SMA: alpha-smooth muscle actin, DSO: Descemet stripping only; IHC: immunohistochemistry; PRK: photorefractive keratectomy; PTK: phototherapeutic keratectomy.

**Table 2:** Clinical studies in the literature.

Study (Year)	Patients/Eyes (N)	Indication	Interval Between the Inciting Event and Initiation of Losartan	Losartan Formulation & Dose	Treatment Duration	Visual Acuity Outcomes	Objective Imaging Outcomes	Safety
Domene-Hickman et al., *Ther Adv Ophthalmol*, 2025. [23]	17 patients/19 eyes	Corneal scarring secondary to infectious keratitis (herpetic/bacterial/fungal; n = 12), hydrops (n = 3), interstitial keratitis (n = 2), chemical burn (n = 1), and pterygium surgery (n = 1).	41.37 ± 78.97 months after the initial injury	1 mg/mL; 6×/day	3 months	Improvement in 9 eyes (47%) gaining ≥2 lines; partial improvement in 5 eyes (26%) gaining 1 line; no change in 4 eyes (21%); worsening in 1 eye (5%) losing ≥2 lines.	Decrease in total corneal densitometry (34.02 → 31.76 GSU), total central densitometry excluding the peripheral 10–12 mm zone (29.51 ± 8.57 → 27.12 ± 6.32 GSU), and posterior corneal densitometry (16.94 ± 5.31 → 15.26 ± 3.40 GSU); all *p* < 0.05.	No discontinuations due to adverse effects
Burgos-Blasco & Moloney, *Cornea,* 2024. [24]	7 patients/8 eyes	Post-PRK haze (n = 3), post-PRK–CXL haze (n = 2), post-CXL haze (n = 1), post-adenoviral nummular keratitis (n = 1), corneal fibrosis secondary to chronic edema (n = 1)	34.9 ± 39.6 months after initial injury	0.8 mg/m; 4×/day	1–6 months	Improvement in 5 eyes; no change in 1; worsening in 2	Non-significant trend toward Pentacam densitometry reduction (Friedman *p* > 0.05).	Good tolerance; mild discomfort; adherence issues
Dutra et al., *Case Rep Ophthalmol,* 2025. [28]	3 patients/3 eyes	Herpes-related corneal scarring.	2–7 months.	0.8 mg/mL; 6×/day	4–9 months	Visual acuity improved in all 3 eyes, with substantial gains in 2 cases.	Qualitative improvement on anterior segment OCT imaging. No densitometric data.	No significant adverse events; dosing precautions advised
Rodgers et al., *Cornea*, 2024. [25]	1 eye	Severe post–epi-off CXL haze progressing to stromal scarring	107 days after CXL	0.8 mg/mL; 6×/day	~3 months	VA improved from 20/150 to 20/40; marked haze reduction	Pentacam densitometry reduction (32.2 → 27.9 GSU)	No adverse events reported
Vindel Valle et al., *Arch Soc Esp Oftalmol,* 2025. [26]	1 eye	Post-infectious leucoma with neovascularization.	~2 months after the infectious episode.	0.8 mg/mL; 6×/day	3 months	VA improved from hand motion to 20/200	Reduction in leucoma and vessel caliber in slit lamp photographs.	No major adverse events reported
Pereira-Sousa et al., *J Refract Surg,* 2022 [27]	1 eye	Severe subepithelial corneal haze after complicated LASIK	52 days after LASIK	0,8 mg/mL 6×/day	4.5 months	UDVA improved 20/200 → 20/30; CDVA improved 20/30 → 20/25.	Reduction in corneal haze documented by Scheimpflug tomography and anterior segment OCT	No adverse events reported

VA: visual acuity, UDVA: uncorrected distance visual acuity, CDVA: corrected distance visual acuity, PRK: photorefractive keratectomy, CXL: cross-linking, OCT: optical coherence tomography, LASIK: Laser-Assisted In Situ Keratomileusis.

## Data Availability

No new data were created or analyzed in this study.
